# Acute Toxicity, Bioaccumulation and Elimination Rate of Deltamethrin and Cypermethrin in Crucian Carp (*Carassius auratus*)

**DOI:** 10.3390/biology14040388

**Published:** 2025-04-09

**Authors:** Zhongquan Jiang, Yunyun Ji, Ruikai Xing, Xinyi Xie, Guangxin Yang, Cong Kong, Xiaosheng Shen

**Affiliations:** 1 East China Sea Fisheries Research Institute, Chinese Academy of Fishery Sciences, Shanghai 200090, China; zhongquanjiang@outlook.com (Z.J.); jiyuny2020@163.com (Y.J.); 17720685183@163.com (X.X.); yangpai0717@163.com (G.Y.); kongcong@gmail.com (C.K.); 2 State Environmental Protection Key Laboratory of Environmental Health Impact Assessment of Emerging Contaminants, School of Environmental Science and Engineering, Shanghai Jiao Tong University, Shanghai 200240, China; xrk382937427@sjtu.edu.cn

**Keywords:** deltamethrin, cypermethrin, acute toxicity, bioaccumulation, elimination, *Carassius auratus*

## Abstract

Pyrethroid pesticides like deltamethrin and cypermethrin are widely used in aquaculture, yet their food safety implications remain understudied. This research investigated their acute toxicity, tissue-specific bioaccumulation, and elimination patterns in crucian carp (*Carassius auratus*). Acute toxicity tests determined 96 h LC50 values of 9.68~11.22 ng·mL⁻^1^ (deltamethrin) and 3.12~5.01 ng·mL⁻^1^ (cypermethrin) with 95% confidence intervals. During 8-day exposures to sublethal concentrations (1/10 and 1/100 of LC50), deltamethrin accumulated predominantly in the liver (peak: 21.98 ng·g⁻^1^ at 1.04 ng·mL⁻^1^, standard deviation is 0.064 ng·mL⁻^1^), whereas cypermethrin concentrated in muscle (peak: 9.76 ng·g⁻^1^ at 0.40 ng·mL⁻^1^, standard deviation is 0.138 ng·mL⁻^1^). A 7-day elimination phase revealed faster clearance of low-concentration residues, with >50% removal within 24 h in all tissues. Bioconcentration factors were highest in the liver (36.62 for deltamethrin) and muscle (45.17 for cypermethrin). These results highlight tissue-specific accumulation risks and rapid initial elimination, providing critical data to optimize pesticide-dosing protocols, mitigate ecotoxicological threats, and enhance food safety in aquaculture systems.

## Simple Summary

This study examined how two common pesticides used in fish farming—deltamethrin and cypermethrin—affect crucian carp. These chemicals effectively kill pests but may pose risks to food safety. We tested how toxic these pesticides are to fish and where they build up in their bodies. Results showed that deltamethrin was more toxic than cypermethrin, with lethal concentrations (96-h exposure) of 10.43 ng/mL and 3.95 ng/mL, respectively. When exposed to lower, non-lethal doses for 8 days, deltamethrin accumulated most in the liver, while cypermethrin built up primarily in muscle. After moving fish to clean water, low doses were eliminated faster than high doses. The liver and muscle showed the highest capacity to concentrate these pesticides. Importantly, most pesticide residues were removed from fish tissues within 24 h of clean water exposure. These findings help fish farmers use the pesticides more safely, reducing risks to aquatic life and ensuring safer seafood for consumers in China.

## 1. Introduction

Pyrethroid insecticides can effectively eliminate pests in fruits and vegetables as well as parasites on freshwater fish, making them widely utilized in agriculture and aquaculture [1]. Among them, deltamethrin (C_22_H_19_Br_2_NO_3_) and cypermethrin (C_22_H_19_Cl_2_NO_3_) are the most extensively and commonly used pyrethroids [2]. Deltamethrin, containing an alpha-cyano group, is classified as a type II pyrethroid, whereas cypermethrin is classified as a type I pyrethroid. Both compounds belong to artificially synthesized biomimetic insecticides [3]. In an outdoor pond microcosm experiment, deltamethrin exhibited a degradation half-life ranging from 8 to 48 h after spray drift [4]. Cypermethrin is relatively persistent in water, with a half-life time of less than 1 year. [5]. In aquaculture, deltamethrin and cypermethrin are applied to manage parasites and pests. For example, deltamethrin is employed in salmon farming to control parasitic copepods [6]; Additionally, both deltamethrin and cypermethrin effectively control *Brachionus calyciflorus* [7]. However, food safety issues arising from deltamethrin and cypermethrin residues in aquatic products have recently garnered widespread concern [8,9].

Most pyrethroid pesticides are highly lipophilic insecticides. They can directly enter the gills and blood of fish in water and interfere with the nerve conduction function of non-target organisms through sodium ion channels [10]. Studies have found that long-term exposure and excessive intake of deltamethrin and cypermethrin by zebrafish, rotifers, tilapia, etc., can lead to the release of neurotransmitters and direct damage to the nervous system, such as hindering behaviors like predation and evading natural enemies, and may have an adverse impact on the individual growth in their early life stage [7,11,12,13,14]. In addition, deltamethrin can hinder the development of organisms such as (*Gobiocypris rams*), inhibit the immune function of fish, make fish susceptible to *Pseudomonas fluorescens*, and thereby lead to death [15]; cypermethrin can also affect the movement of aquatic organisms such as carp and affect the normal operation of the immune system [16]. The European Union stipulates that the maximum residue limits of deltamethrin and cypermethrin in fish are 10 μg·kg^−1^ and 50 μg·kg^−1^, respectively [17]. China has now explicitly stipulated that the residue of deltamethrin in fish tissues shall not exceed 30 μg·kg^−1^, and that of cypermethrin shall not exceed 50 μg·kg^−1^ [18]. Despite China’s clear regulations on the use of deltamethrin and cypermethrin, there are still phenomena such as excessive pesticide concentrations and environmental pollution in aquaculture. Huang and others conducted tests on grass carp, crucian carp, carp, and bighead carp in 24 freshwater farms in Northeast China and found that the cypermethrin in grass carp, crucian carp, and carp exceeded the standard [19]. The maximum detected concentrations were 198.19 μg·kg^−1^, 181.73 μg·kg^−1^, and 132.74 μg·kg^−1^, respectively, exceeding the standard of accepted levels. Pyrethroid pesticides are widely used in agriculture and aquaculture due to their efficient insecticidal properties. However, behind this widespread use lies a potential threat to aquatic organisms.

Crucian carp (*Carassius auratus*) is among the most widely cultivated aquaculture species in China. According to the *2024 China Fishery Statistical Yearbook*, crucian carp production in China reached 2.84 million tons in 2023, ranking fifth among freshwater fish species [20]. However, deltamethrin and cypermethrin, two commonly used pyrethroid pesticides, are extensively applied in agriculture and frequently used in crucian carp farming, resulting in unavoidable residues entering aquaculture water [21]. Although research has investigated the impact of various pesticide residues on fish, studies specifically addressing deltamethrin and cypermethrin residues in crucian carp tissues remain limited. Currently, the acute toxicity of these two pesticides in crucian carp tissues remains unclear, their metabolic pathways lack comprehensive investigation, and systematic studies on residual levels in crucian carp are insufficient.

This study investigates the bioaccumulation and elimination patterns of two representative pyrethroid pesticides, deltamethrin and cypermethrin, in crucian carp. The objective is to provide critical data for monitoring pyrethroid residues in freshwater aquaculture, thereby improving product quality and ensuring aquatic food safety.

## 2. Materials and Methods

### 2.1. Experimental Design

Approximately 600 crucian carp (*Carassius auratus*), averaging 100.00 ± 10.00 g, were sourced from the Shanghai Yongchao Aquaculture Professional Cooperative breeding base. Fish were acclimated for 7 days prior to the acute toxicity test and 14 days prior to the bioaccumulation test. Fish were reared under experimental conditions in eight 500-L semi-closed aquaculture tanks containing breeding water continuously aerated for 48 h. Half of the water was replaced daily, and water quality parameters were monitored every day. During acclimation, fish were fed once daily with pellets at a feeding rate of 1~3% of their body weight. Food residues, excreta, and any deceased fish were removed daily, and feeding ceased 24 h before each experiment commenced. Following the guidelines of the MEP and the OECD (2012), water temperature was maintained at 22~25 °C, pH ranged from 7.4 to 8.6, and dissolved oxygen was kept above 8.0 mg·L^−1^ [22,23]. The acute toxicity tests were conducted in glass containers (40 cm × 23 cm × 25 cm), while bioaccumulation and elimination experiments were carried out in larger breeding ponds (185 cm × 140 cm × 100 cm). Containers were filled with varying volumes of breeding water: 100 L for acute toxicity tests and 2000 L for bioaccumulation and elimination tests. Containers were continuously aerated for 24 h prior to fish introduction.

#### 2.1.1. Acute Toxicity Test

According to pre-test results (Table 1), the maximum non-lethal concentration of deltamethrin for crucian carp (*Carassius auratus*) was 8.00 ng·mL^−1^, and the minimum lethal concentration was 16.09 ng·mL^−1^. For cypermethrin, these values were 1.25 ng·mL^−1^ (maximum non-lethal) and 15.28 ng·mL^−1^ (minimum lethal). Consequently, six proportional concentration gradients were established: deltamethrin concentrations at 8.00, 9.20, 10.58, 12.17, 13.99, and 16.09 ng·mL^−1^, and cypermethrin concentrations at 1.25, 2.06, 3.40, 5.61, 9.26, and 15.28 ng·mL^−1^. Additionally, a control group without pesticide exposure was included, and each concentration level had three replicates. Glass tanks containing 100 L of deltamethrin or cypermethrin solutions at various concentrations were prepared, with each tank stocked with 10 crucian carp. Due to the instability of cypermethrin and deltamethrin, a semi-static exposure method was used, replacing solutions every 24 h [7]. Deltamethrin (99.7% purity) and cypermethrin (97.3% purity), purchased from the Shanghai Institute of Measurement and Testing Technology, were dissolved to prepare the test solutions at varying concentrations. Poisoning symptoms and mortality were continuously monitored and recorded during the first 6 h after exposure, and subsequently recorded at intervals of 24, 48, 72, and 96 h.

#### 2.1.2. Bioaccumulation and Elimination Test

Based on the acute toxicity test results, concentrations equivalent to 1/10 and 1/100 of the 96-h LC50 were selected. Concentration groups and a control group were established, each with three replicates. Each breeding pond contained 2000 L of filtered aquaculture water and 60 crucian carp. The experimental solutions were prepared by directly dissolving deltamethrin and cypermethrin standards into the water medium. During the experiment, 50% of the solution was renewed daily to maintain the pesticide concentrations. Fish were fed daily with pellets at 3% of their body weight. Uneaten food, excreta, and deceased fish were removed daily to ensure optimal water quality. Crucian carp were randomly sampled at 0, 1, 2, 4, 6, and 8 days after the start of the bioaccumulation experiment. The water temperature was continuously monitored, and pH and dissolved oxygen were measured regularly to maintain stable water quality conditions.

To investigate the elimination of pesticides, the breeding ponds were refilled with pesticide-free aquaculture water. The temperature of this replacement water was adjusted to approximately 25 °C, consistent with the experimental conditions, and aerated continuously for 72 h before use. Samples were collected on days 9, 10, 12, and 15 after replacing the water.

At each sampling time, three crucian carp were randomly selected from each pond, and blood, liver, kidney, and muscle samples were collected. Blood was drawn using a 5 mL disposable syringe inserted into the heart at the base of the pectoral fin, and immediately transferred into a 5 mL vacuum blood collection tube [24]. Subsequently, liver, kidney, and muscle tissues were separately homogenized, placed into 50 mL centrifuge tubes, and stored at −18 °C until analysis.

### 2.2. Chemical Analysis

The established analytical method was validated using a blank matrix spiking approach. Six concentration levels (1, 5, 10, 20, 50, and 100 ng·mL^−1^) were added to the blank matrices (water and crucian carp tissues), each with six replicates, to determine recovery rates. Recovery rates for deltamethrin in water and crucian carp tissues ranged from 86.3% to 101.8% and 79.4% to 107.5%, respectively, with relative standard deviations of 2.2% to 6.4% and 2.5% to 7.9%, respectively. For cypermethrin, the recovery rates in water and crucian carp tissues ranged from 79.8% to 107.8% and 73.1% to 98.2%, respectively, with relative standard deviations of 2.5% to 6.5% and 1.9% to 7.5%, respectively.

A 10.00 g (±0.01 g) portion of the homogenized sample was accurately weighed into a 50 mL capped centrifuge tube. Then, 10 mL of acetone (J.T.Baker, NJ, USA) was precisely added, followed by vortex mixing for 1 min and ultrasonic extraction for 10 min. Subsequently, 15 mL of sodium chloride solution (Sinopharm Chemical Reagent Co., Ltd., Beijing, China) and 10 mL of n-hexane (J.T.Baker, Phillipsburg, NJ, USA) were added. The mixture was vigorously shaken, extracted, and centrifuged at 3000 rpm for 5 min. The upper n-hexane phase was transferred to another 30 mL capped centrifuge tube. Extraction was repeated once more with an additional 10 mL of n-hexane, and both n-hexane phases were combined. Then, 3 g of anhydrous magnesium sulfate was added, thoroughly vortexed, and centrifuged at 4000 rpm for 5 min. The supernatant was carefully transferred into a glass tube and evaporated under nitrogen flow until nearly dry (remaining volume < 0.5 mL). The residue was dissolved in 10 mL of acetonitrile (saturated with n-hexane; J.T.Baker, USA), transferred to a 30 mL centrifuge tube, ultrasonicated for 1 min, and vortex-mixed for 30 s. Subsequently, 2 mL of n-hexane (saturated with acetonitrile) was added, vortex-mixed, and centrifuged at 4000 r·min^−1^ for 5 min. The upper n-hexane phase was transferred to another 30 mL centrifuge tube, and extraction was repeated once with an additional 10 mL of n-hexane. Both n-hexane extracts were combined. Next, 3 g of anhydrous magnesium sulfate was added, thoroughly vortexed, and centrifuged at 4000 r·min^−1^ for 5 min. The supernatant was then transferred accurately into a glass tube and evaporated under a nitrogen stream until approximately 0.5 mL remained.

The quantification of deltamethrin and cypermethrin residues in crucian carp tissues was performed using gas chromatography-mass spectrometry (TSQ Quantum GC; Thermo Scientific, Waltham, MA, USA) with a DB-5MS capillary column (30 cm × 0.25 mm × 0.25 μm). Chromatographic separation followed a temperature gradient: 80 °C (2 min) → 200 °C at 15 °C·min⁻^1^ → 280 °C at 5 °C·min⁻^1^ (10 min hold), using helium carrier gas (1.2 mL·min⁻^1^). Electron ionization (70 eV) was applied, with injector/ion source temperatures at 250 °C and 230 °C, respectively. Compounds were identified via retention times and spectral matches (NIST 17 library similarity > 85%). Method specificity was confirmed by blank matrix analysis, with no interference observed. Sensitivity was validated with LOD (0.5 ng·g⁻^1^) and LOQ (1.5 ng·g⁻^1^), supported by recovery rates of 79.4–107.5% and RSD < 7.9%, ensuring precision at sub-ppb levels. While optimized for parent compounds, metabolite detection was beyond the method’s scope.

### 2.3. Statistical Analysis

The 96-h LC50 value was calculated using a log-logistic regression model with Genstat (version 20) [25]. Statistical analyses were conducted using IBM SPSS Statistics 25. Normality was assessed using a Shapiro–Wilk test, and variance homogeneity was evaluated using a Levene’s test. For normally distributed data with homogeneous variances, concentration differences among sampling days were analyzed by one-way ANOVA followed by a Tukey’s post hoc test at a 95% significance level. All concentration values are presented as means with their corresponding 95% confidence intervals. Then, we calculated bioconcentration factors (BCFs), which reflect the extent to which a chemical substance accumulates in the tissues of an organism relative to its concentration in the surrounding environment.

BCF:

$$\mathrm{BCF}=\frac{\;\mathrm{Concentration}\;\mathrm{of}\;\mathrm{the}\;\mathrm{substance}\;\mathrm{in}\;\mathrm{the}\;\mathrm{organism}\;\left(\mathrm{ng}/\mathrm{kg}\right)}{\;\mathrm{Concentration}\;\mathrm{of}\;\mathrm{the}\;\mathrm{substance}\;\mathrm{in}\;\mathrm{the}\;\mathrm{water}\;\left(\mathrm{ng}/\mathrm{mL}\right)}$$

## 3. Results

### 3.1. Acute Toxicity Test

Based on observed mortality rates, the 96-h LC50 values for deltamethrin and cypermethrin in crucian carp were 10.43 ng·mL^−1^ and 3.95 ng·mL^−1^, respectively. Mortality rates across different treatment groups are presented in Table 1. Crucian carp exposed to high-concentration deltamethrin (16.09 ng·mL^−1^) exhibited a mortality rate of 100% after 96 h. For lower concentration groups of deltamethrin (8.00 ng·mL^−1^ and 9.20 ng·mL^−1^), the 96-h mortality rates were 0% and 40%, respectively. Similarly, crucian carp exposed to high-concentration cypermethrin (15.28 ng·mL^−1^) displayed a mortality rate of 100% at 96 h. For lower concentration cypermethrin groups (1.25 ng·mL^−1^ and 2.06 ng·mL^−1^), the 96-h mortality rates were 0% and 10%, respectively.

### 3.2. Bioaccumulation Test

**Figure 1:**
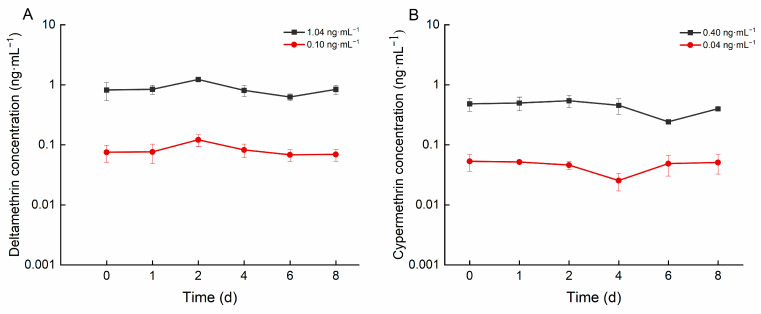
Concentration of deltamethrin and cypermethrin in water of different concentration groups: (**A**): deltamethrin; (**B**): cypermethrin.

As illustrated in Figure 1, the concentrations of deltamethrin in the high- and low-concentration groups ranged from 0.625 to 1.225 ng·mL^−1^ and from 0.068 to 0.121 ng·mL^−1^, respectively. The concentrations of cypermethrin in the high and low concentration groups ranged from 0.241 to 0.542 ng·mL^−1^ and from 0.045 to 0.053 ng·mL^−1^, respectively.

**Figure 2:**
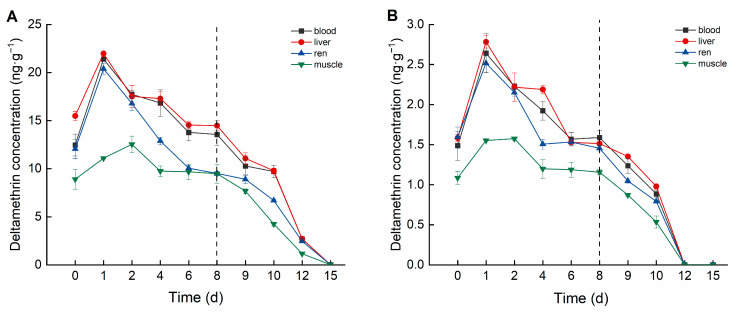
Concentration changes in *C. auratus* tissues under different concentrations of deltamethrin: (**A**) 1.04 ng·g^−1^; (**B**) 0.10 ng·g^−1^. The dotted line is the time when fish were move to water without pesticides.

Deltamethrin accumulation in crucian carp exhibited distinct differences among tissues and varied temporally (Figure 2). In the high concentration group, deltamethrin accumulated in tissues following the order: liver > blood > kidney > muscle. The liver exhibited the highest deltamethrin accumulation, peaking at 21.981 ng·g^−1^ on Day 1, then gradually declining to the lowest concentration (14.475 ng·g^−1^) by Day 8. In blood, deltamethrin concentration peaked at 21.396 ng·mL^−1^ on Day 1, subsequently decreasing and stabilizing between 13.554 and 13.773 ng·mL^−1^ from Day 6 onward. The kidney reached peak accumulation (20.388 ng·g^−1^) on Day 1 and subsequently declined to its lowest concentration (9.499 ng·g^−1^) by Day 8. Muscle tissue reached maximum accumulation (12.528 ng·g^−1^) on Day 2, then gradually decreased and stabilized between 9.475 and 9.738 ng·g^−1^ from Day 4 onward.

In the low-concentration group, the accumulation order of deltamethrin in tissues was consistent with the high concentration group. The liver showed the highest accumulation concentration, peaking at 2.783 ng·g^−1^ on Day 1 and declining to 1.513 ng·g^−1^ by Day 8. The blood reached its maximum accumulation concentration (2.641 ng·mL^−1^) on Day 1, stabilized between 1.568 and approximately 1.588 ng·mL^−1^ from Day 6 onward. The kidney peaked at 2.517 ng·g^−1^ on Day 1 and stabilized between 1.454~1.532 ng·g^−1^ from Day 4 onward. The muscle reached its highest accumulation concentration (1.574 ng·g^−1^) on Day 2 and stabilized between 1.155~1.198 ng·g^−1^ from Day 4 onward.

**Figure 3:**
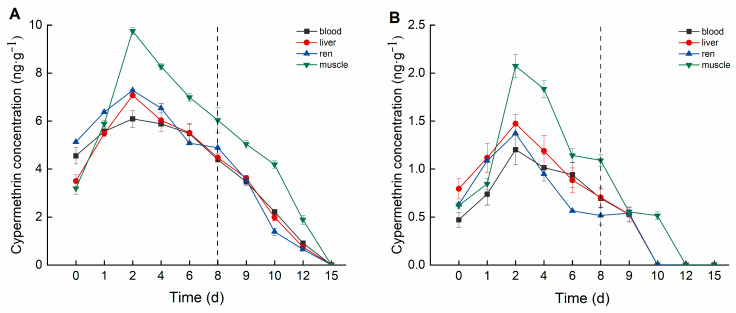
Concentration changes in *C. auratus* tissues under different concentrations of cypermethrin: (**A**): 0.40 ng·g^−1^; (**B**): 0.04 ng·g^−1^. The dotted line is the time when fish were move to water without pesticides.

Cypermethrin accumulation in crucian carp tissues also exhibited distinct differences among tissues and varied over time (Figure 3). In the high concentration group, cypermethrin accumulation followed the order: muscle > kidney > liver > blood. Muscle tissue exhibited the highest cypermethrin accumulation, reaching a peak concentration of 9.755 ng·g^−1^ on Day 2, followed by a decline to 6.035 ng·g^−1^ by Day 8. Kidney tissue reached its peak accumulation (7.275 ng·g^−1^) on Day 2, stabilizing between 4.884 and 5.074 ng·g^−1^ from Day 6 onward. Liver tissue peaked at 7.068 ng·g^−1^ on Day 2, subsequently declining to 4.473 ng·g^−1^ by Day 8. Blood reached a maximum concentration of 6.090 ng·mL^−1^ on Day 2 and then declined to 4.386 ng·mL^−1^ by Day 8.

In the low concentration group, cypermethrin accumulation followed the order: muscle > liver > kidney > blood. Muscle tissue exhibited the highest accumulation, reaching a peak concentration of 2.073 ng·g^−1^ on Day 2, and stabilized between 1.089 and 1.141 ng·g^−1^ from Day 6 onward. Liver tissue reached a peak concentration of 1.472 ng·g^−1^ on Day 2, subsequently declining to 0.703 ng·g^−1^ by Day 8. Kidney tissue peaked at 1.370 ng·g^−1^ on Day 2, stabilizing between 0.514 and 0.562 ng·g^−1^ from Day 6 onward. Blood reached its maximum concentration of 1.201 ng·mL^−1^ on Day 2, then gradually decreased to 0.693 ng·mL^−1^ by Day 8.

### 3.3. Metabolic Elimination Test

**Figure 4:**
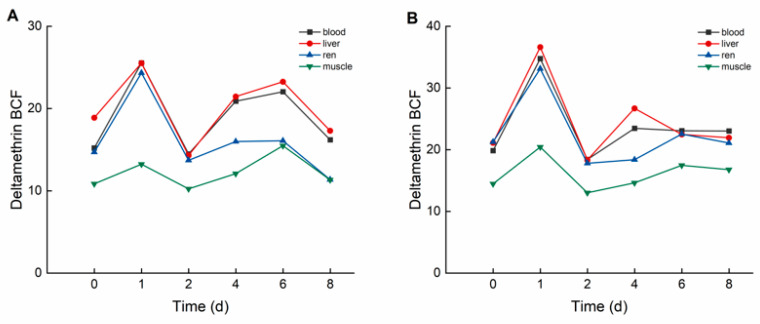
BCF of *C. auratus* tissues at different concentrations of deltamethrin: (**A**): 1.04 ng·g^−1^; (**B**): 0.10 ng·g^−1^.

During the elimination period, deltamethrin and cypermethrin were rapidly eliminated from crucian carp tissues, with concentrations decreasing to relatively low levels. As illustrated in Figure 4, in the high concentration group, the bioconcentration factors (BCFs) of deltamethrin followed the order: liver > blood > kidney > muscle. The maximum BCF values of deltamethrin were 25.514 (liver), 25.532 (blood), 24.329 (kidney), and 15.474 (muscle). In the low concentration group, BCF values of deltamethrin followed the same order, with maximum values of 36.618 (liver), 34.750 (blood), 33.119 (kidney), and 20.421 (muscle).

**Figure 5:**
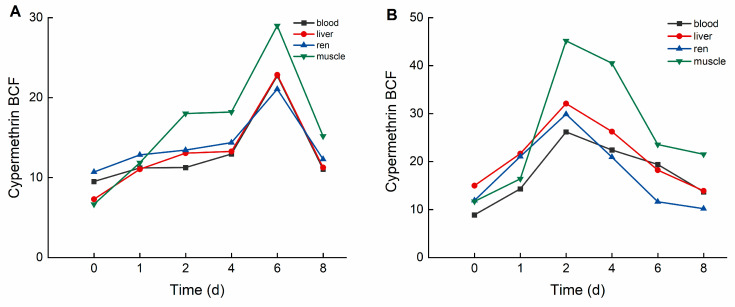
BCF of *C. auratus* tissues at different concentrations of cypermethrin: (**A**): 0.40 ng·g^−1^; (**B**): 0.04 ng·g^−1^.

As illustrated in Figure 5, in the high concentration group, the bioconcentration factors (BCFs) of cypermethrin followed the order: muscle > liver > kidney > blood. The maximum BCF values for cypermethrin were 28.992 (muscle), 22.846 (liver), 21.054 (kidney), and 22.714 (blood). In the low concentration group, the BCF values of cypermethrin followed the same order, with maximum values of 45.166 (muscle), 37.075 (liver), 29.847 (kidney), and 26.166 (blood).

## 4. Discussion

### 4.1. Acute Toxicity

Crucian carp are highly sensitive to pesticides, likely due to their efficient pesticide absorption through the gills and the high susceptibility of their nervous system to pyrethroids [26]. Pyrethroids are neurotoxic agents and research has shown that they disrupt voltage-gated channels, causing neurological dysfunction, metabolic imbalance, and protein inactivation [27]. In this study, crucian carp exposed to pyrethroids exhibited neurotoxic symptoms, including lateral turning, intermittent rapid swimming, and collisions with aquarium walls.

Pyrethroids exhibit high toxicity to fish [28,29]. De Souza et al. reported that deltamethrin exhibited high toxicity to five fish species (*Carnegiella strigata*, *Colossoma macropomum*, *Corydoras schwartzi*, *Hemigrammus rhodostomus*, and *Paracheirodon axelrodi*), with 96-h LC50 values ranging from 6.69 to 23.63 μg/L, except for C. *schwartzi*, which had a significantly higher LC50 of 183.51 μg/L [30]. Abubakar et al. determined that cypermethrin had a 96-h LC50 of 12 ng·mL^−1^ for tilapia, categorizing it as “extremely toxic” [29]. In this study, deltamethrin and cypermethrin were both classified as “extremely toxic” to crucian carp, with 96-h LC 50 values of 10.43 ng·mL^−1^ and 3.95 ng·mL^−1^, respectively. Combining previous research with the findings of this study, it is evident that pyrethroids pose a significant toxic threat to freshwater fish. Therefore, strict regulation of deltamethrin and cypermethrin use in freshwater aquaculture is necessary to mitigate risks to fish populations.

### 4.2. Environmental Factors

During the exposure period, deltamethrin and cypermethrin concentrations in water fluctuated significantly across treatment groups and replicates (Figure 1). This fluctuation may result from multiple interacting factors. First, the sampling process is intrusive and inevitably disrupts the water’s chemical composition and concentration distribution. Second, the mortality of some crucian carp during the experiment reduced the system’s biomass, disrupting the equilibrium of material exchange between the aquatic environment and organisms and consequently affecting pesticide concentrations in the water. Additionally, the high uptake efficiency of crucian carp for deltamethrin and cypermethrin, coupled with the 24-h renewal interval, was insufficient to offset the fish’s absorption capacity. Consequently, pesticide concentrations in the water failed to stabilize at the preset nominal levels.

### 4.3. Bioenrichment and Elimination

In this study, deltamethrin and cypermethrin accumulated in various crucian carp tissues following aqueous exposure, exhibiting distinct bioaccumulation patterns across tissues. In the deltamethrin accumulation experiment, the liver exhibited the highest accumulation, whereas muscle had the lowest. This may be attributed to the liver’s role as the primary detoxification and metabolic organ, responsible for biotransforming and metabolizing xenobiotics into smaller, excretable molecules [31]. Conversely, in the cypermethrin accumulation experiment, muscle exhibited the highest accumulation, whereas blood had the lowest. This may be attributed to cypermethrin’s strong lipophilicity, along with the relatively simple composition and higher lipid content of muscle tissue [32]. Therefore, future studies should focus on monitoring deltamethrin residues in the liver. Given that muscle is the primary edible part of crucian carp, it should be the key target for cypermethrin residue detection in fish. This study also found that at lower deltamethrin concentrations, the bioconcentration factors (BCFs) in blood, liver, kidney, and muscle were higher than in the high-concentration group, a trend also observed for cypermethrin. These findings suggest that pyrethroid pesticides at lower concentrations are more likely to bioaccumulate in organisms.

The elimination trends of deltamethrin and cypermethrin in crucian carp tissues indicated that lower concentrations led to faster elimination. During the deltamethrin elimination phase, pesticide concentrations in the blood, liver, kidney, and muscle of the high-concentration group declined below the detection limit by Day 15, whereas in the low-concentration group, this occurred by Day 12. During the cypermethrin elimination phase, pesticide concentrations in all tested tissues of the high concentration group dropped below the detection limit by Day 15. In the low concentration group, pesticide concentrations in the blood, liver, and muscle fell below the detection limit by Day 12, while the kidney reached this level by Day 10. The kidney’s faster elimination of cypermethrin may be attributed to its lower accumulation capacity and its role as a detoxification organ, facilitating more efficient clearance [33].

### 4.4. Outlook

China’s aquaculture industry is advancing toward intensification, large-scale production, and modernization, resulting in a growing demand for agricultural and veterinary pesticides. Consequently, the presence of residual agricultural and veterinary pesticides in aquaculture environments will remain a critical concern. Future environmental impact studies should focus on investigating the half-life and degradation rates of these pesticides in aquaculture environments, along with the migration and transformation patterns of their metabolic products. This will offer comprehensive insights for evaluating the environmental risks posed by agricultural and veterinary pesticide residues. Additionally, this study demonstrates that residual pesticides in aquaculture environments can bioaccumulate in organisms. Future research should explore the mechanisms of pesticide bioaccumulation in organisms, along with their effects on the intestinal microbial community structure and the prevalence of antibiotic resistance genes. These investigations will provide essential data for assessing the biological risks associated with agricultural and veterinary pesticides.

According to the experimental results, the highest concentration of deltamethrin in the high group reached 21.98 ng·g^−1^, which meets Chinese standards but does not meet EU standards. Therefore, fish intended for sale in Europe cannot be treated with this concentration of deltamethrin, but they can be raised in water with a concentration of 0.1 ng·g^−1^, as the highest concentration in the low group is 2.78 ng·g^−1^. The highest concentration of cypermethrin reached 9.76 ng·g^−1^, which meets both Chinese and EU standards, and fish can be raised in a water solution with a concentration of 0.4 ng·g^−1^ of cypermethrin.

## 5. Conclusions

This study investigated the typical pyrethroid pesticides deltamethrin and cypermethrin as target pollutants, using crucian carp (*Carassius auratus*) as the model organism. A semi-static experimental method was employed to examine the acute toxicity, bioaccumulation, and elimination rates of these pesticides in crucian carp. The results indicate that deltamethrin and cypermethrin concentrations in crucian carp tissues peaked within 1–2 days. The bioconcentration factors (BCFs) of deltamethrin followed the order: liver > blood > kidney > muscle, whereas for cypermethrin, the order was muscle > liver > kidney > blood. After transferring the fish to clean water, most accumulated deltamethrin and cypermethrin were eliminated from all tissues within 24 h. These findings offer critical insights into the transmission and bioaccumulation of these pesticides in the food chain, providing essential data for developing scientific guidelines on pesticide use and ensuring food safety.

## Figures and Tables

**Table 1:** Experimental results of acute toxicity of deltamethrin and cypermethrin to crucian carp.

Pesticides	Test Concentration (ng·mL^−1^)	Mortality (%)				Maximum Total Viability Concentration (ng·mL^−1^)	Minimum Total Lethal Concentration (ng·mL^−1^)	96 h-LC_50_ (ng·mL^−1^)	95% Confidence Interval (ng·mL^−1^)
		24 h	48 h	72 h	96 h				
Deltamethrin	0.00	0 ± 0	0 ± 0	0 ± 0	0 ± 0	8.00	16.09	10.43	9.68~11.22
	8.00	0 ± 0	0 ± 0	0 ± 0	0 ± 0				
	9.20	7 ± 5	20 ± 8	33 ± 5	40 ± 8				
	10.58	10 ± 8	10 ± 8	23 ± 5	50 ± 8				
	12.17	20 ± 8	30 ± 8	63 ± 5	83 ± 5				
	13.99	53 ± 5	77 ± 5	83 ± 5	87 ± 5				
	16.09	53 ± 5	73 ± 5	90 ± 8	97 ± 5				
Cypermethrin	0.00	0 ± 0	0 ± 0	0 ± 0	0 ± 0	1.25	15.28	3.95	3.12~5.01
	1.25	0 ± 0	0 ± 0	0 ± 0	0 ± 0				
	2.06	3 ± 5	3 ± 5	10 ± 8	13 ± 5				
	3.40	13 ± 5	20 ± 8	23 ± 5	40 ± 8				
	5.61	40 ± 8	73 ± 5	77 ± 5	80 ± 8				
	9.26	40 ± 8	73 ± 9	87 ± 12	90 ± 8				
	15.28	63 ± 5	90 ± 8	93 ± 5	97 ± 5				

## Data Availability

No new data were created.

## Abbreviations

The following abbreviations are used in this manuscript:

BCF	Bioconcentration Factors
LC50	Lethal Concentration 50
GC-MS	Gas Chromatography-Mass Spectrometry
